# The dose-effect and clinical prediction of the longest apnea duration driving the decrease of blood oxygen: a large sample OSA study with 34-second and 52-second cut-off values was established

**DOI:** 10.3389/fphys.2025.1691994

**Published:** 2026-01-06

**Authors:** Xiaobo Zhou, Simin Gao, Ping Zeng, Lin Li

**Affiliations:** 1 Provincial Center for Mental Health, Sichuan Provincial People’s Hospital, University of Electronic Science and Technology of China, Sichuan, Chengdu, China; 2 Department of Otolaryngology-Head & Neck Surgery & Sleep Medicine Center, West China School of Public Health and West China Fourth Hospital, Sichuan University, Sichuan, Chengdu, China

**Keywords:** dose-response relationship, hypoxia, longest apnea duration, lowest oxygen saturation, obstructive sleep apnea, predictive model

## Abstract

**Objective:**

To quantify the relationship between the longest apnea duration (LAD) and the lowest oxygen saturation (LSaO_2_) in patients with obstructive sleep apnea (OSA) and to develop a predictive model for the risk of LSaO_2_ decline.

**Methods:**

A total of 1716 OSA patients were enrolled and grouped by severity (236 non-OSA, 395 mild, 365 moderate, and 720 severe). Multiple linear regression was used to analyze the dose-effect relationship between LAD and LSaO_2_. A logistic regression model was developed to predict LSaO_2_ grade, with the dataset partitioned into a training set (n = 1,372) and a testing set (n = 344) using random sampling.

**Results:**

(1) For every 1-s increase in LAD, LSaO_2_ decreased by 0.280% (95% *CI*: −0.291%∼-0.269%) in a univariate model and still decreased by 0.183% (95% *CI*: −0.197%∼-0.170%) after adjusting for sex, age, BMI, and AHI; (2) Critical points were identified: LSaO_2_ was 85% when LAD was 34.20 s and 80% when LAD was 52.07 s; (3) The predictive model showed excellent identification performance for severe (AUC = 0.93) and moderate-severe LSaO_2_ (AUC = 0.96).

**Conclusion:**

The study first quantifies the dose-response relationship between LAD and LSaO_2_ and establishes relevant clinical thresholds. The developed model can accurately identify patients at risk of severe and moderate-severe hypoxia, offering a new tool for individualized intervention.

## 1 Introduction

Obstructive sleep apnea (OSA) is a heterogeneous disorder and the second most common sleep disorder in adults after insomnia. It is characterized by recurrent episodes of apnea and hypopnea during sleep, involving repetitive events of complete (apnea) or partial (hypopnea) collapse of the pharyngeal airway. These events can lead to intermittent hypoxemia, hypercapnia, sleep fragmentation, and cardiac sympathetic alterations, among others (Falla et al., 2023; Heinzer et al., 2015). As the most prevalent type of sleep-disordered breathing, OSA accounts for approximately 80% of all sleep apnea syndrome cases (Gresova et al., 2023). The core pathological injury in OSA stems from the intermittent hypoxia induced by these respiratory events. Current clinical practice relies on the apnea-hypopnea index (AHI) to assess disease severity. However, AHI solely quantifies event frequency and fails to reflect the duration of individual apneic events and the severity of associated oxygen desaturation (Wang et al., 2025). This limitation leads to two major clinical dilemmas: (1) risk misclassification, where patients with the same AHI (e.g., 30 events/hour) classified as severe exhibit substantial differences in organ damage risk depending on apnea duration (e.g., 10 s vs. 60 s) (Punjabi, 2016); and (2) a therapeutic gap, as persistent hypoxemia is observed in some patients achieving CPAP treatment targets (AHI <5 events/hour), significantly increasing their risk of cardiovascular events (Peker et al., 2016).

Unlike the AHI, which only counts respiratory events, apnea duration directly quantifies both single-event and cumulative hypoxia exposure, aligning more with the pathophysiology of hypoxic injury and offering a more direct and sensitive reflection of actual hypoxia levels. Research shows that longer apnea/hypopnea events are linked to lower nocturnal minimum oxygen saturation and more severe hypoxia (Wang et al., 2025; Ma et al., 2024). While the longest apnea duration (LAD) indicates the maximum respiratory event duration, its quantitative link to minimum SaO_2_ (LSaO_2_) remains unclear. Although physiological studies have shown that there is generally a linear relationship between the duration of apnea and the degree of desaturation under controlled conditions, this relationship can be regulated by sleep stage, baseline lung volume, and metabolic factors (Badran and Gozal, 2025; Prabhakar and Kline, 2002; Richardson et al., 2009). In addition, there is a lack of large-sample validation, and key clinical thresholds (such as the critical point of LAD leading to LSaO_2_ < 85%) are often left blank.

This study proposes the “LAD-driven LSaO_2_ desaturation” dose-effect hypothesis and achieves three key advances through a large-scale investigation: (1) Quantifying the dose-effect relationship: Establishing a linear equation for LAD-LSaO_2_ after adjusting for confounders; (2) Identifying critical clinical thresholds: Discovering two hypoxemia-alert thresholds at 34 s and 52 s; and (3) Developing a predictive tool: Creating an LAD-based model for predicting LSaO_2_ (AUC >0.93). This work will facilitate a paradigm shift in OSA management from a “frequency-oriented” approach (AHI) to an “intensity-oriented” approach (LAD). This shift echoes the growing recognition that metrics capturing the “intensity” or “hypoxic burden” of OSA—such as the total area under the desaturation curve—provide a more pathophysiologically relevant risk stratification than AHI alone. Our study contributes to this paradigm by focusing on LAD, a single, readily available metric that serves as a potent driver of acute, profound hypoxic episodes, a key component of the overall hypoxic burden.

## 2 Materials and methods

### 2.1 Research design and patients

A total of 1716 patients who presented to the Sleep Medicine Center of West China Fourth Hospital, Sichuan University, with symptoms such as snoring and witnessed apnea between January 2023 and December 2024 and underwent overnight polysomnography (PSG) were enrolled. Participants were categorized into four groups based on the American Academy of Sleep Medicine (AASM) criteria (Berry et al., 2012): non-OSA group (AHI <5 events/hour), mild group (5 events/hour ≤ AHI ≤15 events/hour), moderate group (15 events/hour < AHI ≤30 events/hour), and severe group (AHI >30 events/hour).

Inclusion Criteria: (1) Age between 18 and 70 years; (2) Complete clinical data, including gender, age, body mass index (BMI), and sleep monitoring parameters; (3) Total PSG monitoring duration ≥7 h. Exclusion Criteria: (1) History of prior OSA treatment (e.g., oral appliance therapy, OSA-related surgery, continuous positive airway pressure [CPAP] therapy); (2) Presence of other severe comorbidities potentially affecting sleep architecture or respiratory function, including respiratory, cardiovascular, or neurological diseases or malignancies, as well as special populations such as pregnant or lactating women.

### 2.2 Definition of key variables

AHI is the average number of apneas and hypopneas per hour. LAD is the longest apnea event duration during the night. LSaO_2_ is the lowest pulse oxygen saturation during the night. PSG automatically analyzes these metrics, which are then manually checked by technicians.

### 2.3 Polysomnography

Overnight polysomnography was performed on all patients using the SOMNOscreen™ plus PSG + system. Recorded parameters included oronasal airflow, oxygen saturation (SpO_2_), electroencephalography (EEG), electrooculography (EOG), submental electromyography (EMG), thoracic and abdominal respiratory effort, body position, and tibialis anterior EMG. Patients were instructed on the day of monitoring to avoid napping and to refrain from consuming coffee, tea, alcohol, or other beverages known to interfere with sleep. They were also asked to maintain clean facial skin and fingers to facilitate signal acquisition. Upon arrival at the hospital, all patients were instrumented by trained sleep technicians for overnight PSG monitoring. The total sleep monitoring duration was required to be ≥7 h. Prior to the PSG study, height and weight were measured for each patient, and BMI was calculated.

### 2.4 Research methods

Before developing the machine learning model, the following data preprocessing steps were performed. First, the gender categorical variable was converted to a numerical variable (male = 0, female = 1) for model compatibility. Second, feature variables were standardized to have a zero mean and unit variance. Third, synthetic minority oversampling technique (SMOTE) was applied to address data imbalance. The standardized dataset was used for model training and prediction.

The preprocessed dataset was split into training and testing sets in a 8:2 ratio. Specifically, 80% of the data were allocated for training the model, while 20% were reserved for testing and validating its performance. Logistic regression is selected as the main machine learning algorithm.

### 2.5 Statistical analyses

The Kolmogorov-Smirnov method was used to test the normality. The quantitative data did not satisfy the normal distribution, and the quartile M (P25, P75) was used to describe it. The qualitative data were described by percentage (%). The Kruskal–Wallis H test was used for inter-group comparisons of quantitative data. For qualitative data, inter-group comparisons were conducted using the Pearson chi-square test or Fisher’s exact probability test. The dose-effect relationship between LAD and LSaO_2_ was analyzed by linear regression analysis. Unless otherwise specified, all tests were two-tailed with a significance level of α = 0.05. Data imputation, standardization, splitting, model building, model evaluation and comparison, as well as the analysis of the relationship between LAD and LSaO2 and result plotting, were implemented using the Python 3.11 scikit-learn and matplotlib libraries. The training set and test set were split in a ratio of 8:2.

## 3 Results

### 3.1 Patient baseline characteristics

This study included 1716 participants, comprising 236 with non-OSA OSA, 395 with mild OSA, 365 with moderate OSA, and 720 with severe OSA, with severe OSA patients accounting for 42.0%. Among the 1716 participants, 1,370 were male (79.8%). The mean age of patients was 42.0 (34.0, 51.0) years, and the mean BMI was 25.6 (23.5, 27.8) kg/m^2^. Demographic characteristics and sleep monitoring results varied among patients with different OSA severities. As the condition worsened, AHI and LAD progressively increased, whereas LSaO_2_ gradually decreased (*p* < 0.001) (Table 1).

**TABLE 1:** Baseline characteristics of 1716 patients.

Variables	All (n = 1716)	Non-OSA group (n = 236)	Mild group (n = 395)	Moderate group (n = 365)	Severe group (n = 720)	*p*
Male/%	1,370 (79.8)	151 (64.0)	270 (68.4)	295 (80.8)	654 (90.8)	<0.001
Age/yr	42.0 (34.0, 51.0)	38.0 (26.0, 47.0)	39.0 (31.0, 48.0)	44.0 (35.0, 54.0)	44.0 (36.0, 53.0)	<0.001
BMI/(kg/m^2^)	25.6 (23.5, 27.8)	24.0 (21.6, 27.0)	24.3 (22.3, 26.4)	25.5 (23.7, 27.4)	26.8 (25.0, 28.7)	<0.001
AHI/h^-1^	22.9 (8.8, 47.1)	1.5 (0.8, 3.0)	8.9 (6.5, 11.6)	20.8 (17.6, 24.9)	50.9 (40.0, 59.7)	<0.001
LAD/s	43.5 (30.0, 63.0)	19.0 (10.3, 24.0)	32.0 (25.0, 41.0)	43.0 (35.0, 56.0)	63.0 (50.0, 82.0)	<0.001
LSaO_2_/%	83.0 (76.0, 88.0)	91.0 (89.0, 93.0)	88.0 (86.0, 90.0)	83.0 (79.0, 86.0)	75.0 (68.0, 81.0)	<0.001

### 3.2 The dose-effect relationship between LAD and LSaO_2_

In the univariate model, the regression coefficient B of LAD was −0.280, with a 95% CI of (−0.291, −0.269), indicating a negative correlation between LAD and LSaO_2_. This means that for every 1-s increase in LAD, LSaO_2_ decreased by an average of 0.280 percentage points without considering other variables. In the adjusted model, controlling for gender, age, BMI, AHI, and other variables, the regression coefficient B of LAD was −0.183, with a 95% CI of (−0.197, −0.170). Despite the reduced regression coefficient, LAD remained negatively correlated with LSaO_2_. After controlling for gender, age, BMI, AHI, etc., each 1-s increase in LAD was associated with an average 0.183-percentage-point decrease in LSaO_2_ (Table 2).

**TABLE 2:** Linear regression analysis between LAD and LSaO_2_.

Variables	Single-factor model			Adjustment model		
	*B*	*95%CI*	*p*	*B*	*95%CI*	*p*
LAD	−0.280	(-0.291, −0.269)	<0.001	−0.183	(-0.197, −0.170)	<0.001
Gender				−0.932	(-1.595, −0.268)	0.006
Age/yr				−0.024	(-0.046, −0.002)	0.029
BMI/(kg/m^2^)				−0.516	(-0.596,-0.436)	<0.001
AHI/h^-1^				−0.153	(-0.170, −0.135)	<0.001

### 3.3 The ability of LAD to predict the degree of LSaO_2_

We randomly split 1716 samples into a training set (n = 1,372) and a testing set (n = 344) according to 8:2. The two groups showed good consistency in the distribution of AHI severity (Table 3), and both showed the same distribution pattern (severe > mild > moderate > non-OSA). The maximum difference in the percentage distribution of the training set and test set under each severity was only 3.7%.

**TABLE 3:** Distribution of training set and test set in AHI severity.

AHI severity	Total data set (n = 1716)	Training set (n = 1,372)	Testing set (n = 344)
Non-OSA	234 (13.6%)	177 (12.9%)	57 (16.6%)
Mild	394 (23.0%)	325 (23.7%)	69 (20.1%)
Moderate	367 (21.4%)	300 (21.9%)	67 (19.5%)
Severe	721 (42.0%)	570 (41.5%)	151 (43.9%)

**FIGURE 1:**
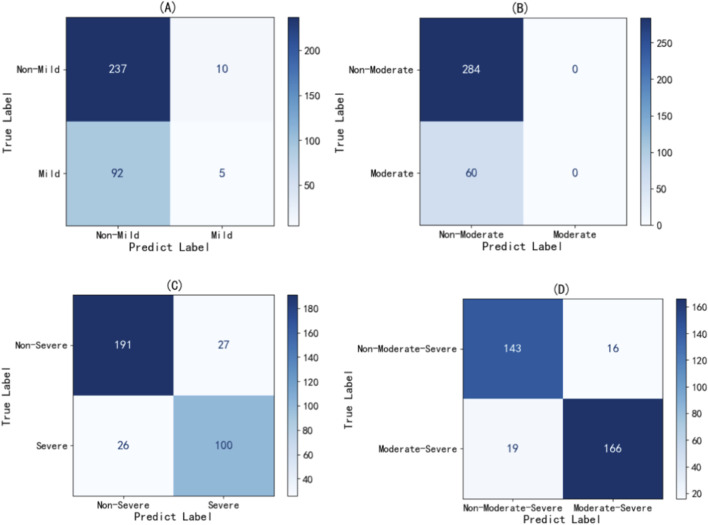
Confusion matrix of prediction model based on Logistic regression in LSaO_2_ test set with different severity. **(A)** Mild LSa02 **(B)** Moderate LSa02 **(C)** Severe LSa02 180 **(D)** Moderate-Severe LSa02.

**FIGURE 2:**
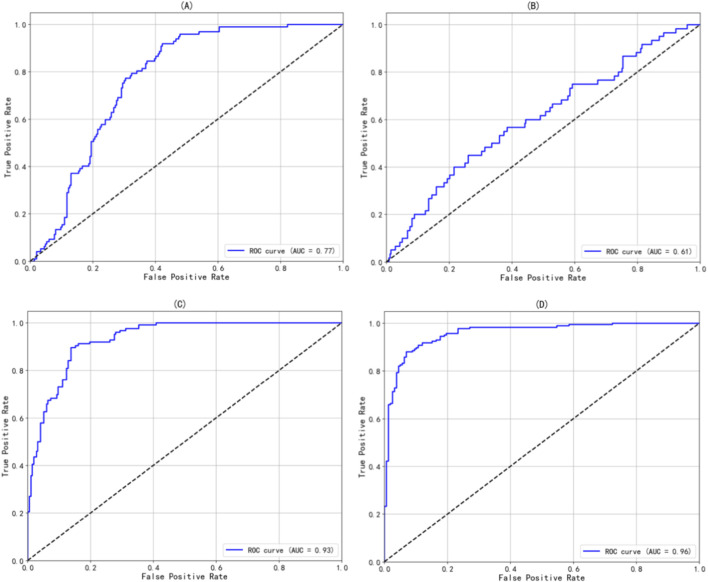
The ROC curve of the prediction model based on Logistic regression in different severity of LSaO_2_. **(A)** Mild LSa02 **(B)** Moderate LSa02 **(C)** Severe LSa02 180 **(D)** Moderate-Severe LSa02.

In this study, LSaO_2_ levels were categorized into four groups: normal (LSaO_2_ ≥90%), mild (85%–90%), moderate (80%–85%), and severe (<80%). A Logistic regression model was used to evaluate LAD’s predictive capacity for different LSaO_2_ severities. The model was developed using a training set (n = 1,372) and assessed on a testing set (n = 344). Figure 1's confusion matrix offers a visual overview of the classification accuracy for mild, moderate, severe, and combined moderate-severe LSaO_2_, showing good performance in severe and combined moderate-severe categories. As indicated in Figure 2, the ROC curve analysis yielded AUC values of 0.77, 0.61, 0.93, and 0.96 for mild, moderate, severe, and combined moderate-severe LSaO_2_ predictions, respectively, highlighting the model’s excellent ability to distinguish severe and moderate-severe LSaO_2_ cases.

### 3.4 The linear relationship between LAD and LSaO_2_

**FIGURE 3:**
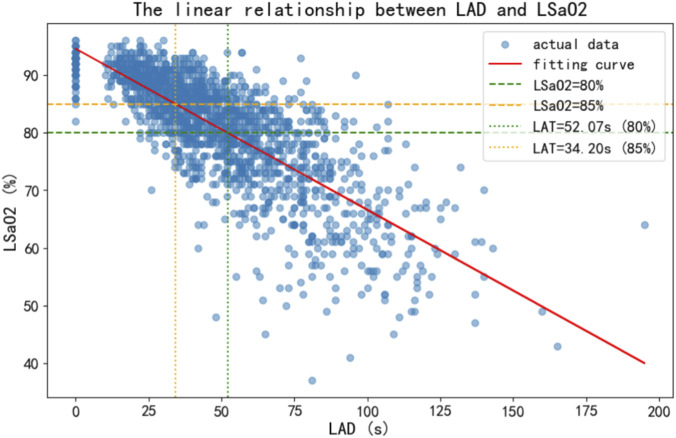
Linear relationship between LAD and LSaO_2_.

To visually illustrate the relationship between LAD and LSaO_2_, this study employed Matplotlib to generate scatter plots with fitted regression lines. The scatter plots display the actual data points, reflecting the distribution of LSaO_2_ across varying apnea durations. The fitted lines represent predictions from linear regression models, demonstrating the linear association between the variables. Additionally, two horizontal reference lines were incorporated at the LSaO_2_ thresholds of 85% and 80% to facilitate visual assessment of LAD’s impact on LSaO_2_. As shown in Figure 3, an LAD of 34.20 s corresponded to an LSaO_2_ of 85%, while an LAD of 52.07 s corresponded to an LSaO_2_ of 80%.

## 4 Discussion

This study analyzed 1716 OSA patients to quantify the dose-response relationship between LAD and LSaO_2_ for the first time, determining critical LAD thresholds of 34 s (LSaO_2_ = 85%) and 52 s (LSaO_2_ = 80%). These findings enhance our understanding of OSA’s pathophysiology and offer valuable guidance for clinical risk stratification and therapeutic strategies.

Our dose-response model demonstrated that for every 1-s increase in LAD, LSaO_2_ decreased by an average of 0.183 percentage points (95% *CI*: −0.197 to −0.170). This linear relationship remained statistically significant after adjusting for confounders, including AHI and BMI. These results provide compelling support for the emerging theory (Oksenberg and Leppanen, 2023a; Oksenberg and Leppanen, 2023b; Kulkas et al., 2017) that respiratory event duration is a core driver of hypoxic severity. A primary determinant of desaturation event duration, depth, and area is the duration of apnea and hypopnea events. Whereas the traditional AHI—the gold standard for OSA diagnosis—quantifies event frequency while ignoring temporal dimensions, this approach results in substantial heterogeneity in hypoxic burden among patients with identical AHI values. Specifically, within the same OSA severity stratum, longer apnea-hypopnea durations and deeper desaturations may carry distinct clinical implications compared to briefer, shallower events (Yilmaz Durmaz and Gunes, 2020).

Although prior research has addressed the association between respiratory event duration and hypoxia, the predominant focus has been on averaged temporal measures (e.g., total event duration per hour (Wang et al., 2025; Ma et al., 2024; Sarac and Afsar, 2020)) or inter-event variations (e.g., differential oxygen reduction efficacy between hypopneas and apneas (Kulkas et al., 2017)). Yılmaz et al. (Yilmaz Durmaz and Gunes, 2020; Muraja-Murro et al., 2012) demonstrated that among respiratory events, obstructive apnea duration exhibits the strongest correlation with oxygen desaturation. By concentrating on the pivotal metric of “single longest apnea duration (LAD),” this study establishes individual LAD episodes as predictors of hypoxia severity, thereby augmenting the pathophysiological framework through event intensity—prolonged apneas induce sustained ventilatory arrest, accelerating depletion of alveolar oxygen reserves. This finding elucidates the clinical paradox wherein certain patients with high AHI exhibit mild hypoxia (shorter events) while others with low AHI develop severe hypoxia (longer events), resonating with Oksenberg et al.'s (Oksenberg and Leppanen, 2023a; Oksenberg and Leppanen, 2023b) theory of “distinct pathological significance of extended respiratory events.” Our work further reveals a unique mechanism for acute profound hypoxia: protracted apneas exacerbate desaturation through prolonged tissue hypoxia exposure and compromised reoxygenation intervals (Badran and Gozal, 2025), a process potentially conferring preferential injury to hypoxia-vulnerable organs (e.g., cardiocerebrovascular systems) (Martinez-Garcia et al., 2023). Clinical evidence (Sufioglu et al., 2012; Li et al., 2011) indicates nasal surgery reduces apnea-hypopnea durations and improves quality of life/daytime somnolence without altering AHI. Moreover, animal studies (Wu et al., 2019) establish that apnea-hypopnea duration correlates strongly with vascular inflammation, endothelial dysfunction, and hypertension; longer events (accompanied by deeper desaturations) provoke heightened systemic inflammation, endothelial impairment, and blood pressure elevation independent of AHI.

Our study overcomes the limitations of AHI and mean event duration (Sarac and Afsar, 2020; Zhan et al., 2018) by focusing on single-peak duration as a core hypoxia predictor for the first time. This approach aligns with clinical reality. For instance, A long time apnea may directly cause myocardial ischemia (e.g., ST-segment depression), while short-term frequent events can be compensated for to alleviate hypoxic damage (Oksenberg and Leppanen, 2023a). This shift echoes the new trend of assessing OSA based on “frequency to severity.” (Martinez-Garcia et al., 2023) Moreover, the linear regression model (*B* = −0.183) enables clinicians to quantify patient-specific hypoxia risks, offering an advantage over traditional AHI stratification. It is particularly useful for high-risk groups with non-severe AHI but long-duration apneas (e.g., AHI = 15 events/hour but LAD >50 s). The thresholds of 34 s (LSaO_2_ = 85%) and 52 s (LSaO_2_ = 80%) carry multifaceted clinical implications: (1) In-home sleep testing, these cutoffs can be integrated into reports to automatically flag high-risk hypoxic events; (2) For patients with prolonged LAD, CPAP pressure titration should be prioritized over behavioral interventions alone; (3) LAD may serve as an early warning signal for cardiovascular complications; (4) Longer LAD before upper airway surgery may indicate poor efficacy, and OSA patients with longer apnea duration are more likely to suffer from the risk of surgical failure (Suen et al., 2019; Bostanci et al., 2016). Bostanci et al. (Bostanci et al., 2016) identified associations between surgical failure and multiple parameters (mean obstructive apnea duration >26.75 s, total apnea duration, minimum SaO_2_, mean SaO_2_, mean O_2_ desaturation, and oxygen desaturation index), yet only mean obstructive apnea duration >26.75 s remained an independent predictor of adverse outcomes after multivariable adjustment [*OR* (95% *CI*) = 3.92 (1.08-14.17), *p* = 0.041]. The thresholds of 34 s (LSaO_2_ = 85%) and 52 s (LSaO_2_ = 80%) offer clear reference points for identifying patients approaching the critical decompensation point.

Despite the significant value of the results, the following limitations should be carefully considered: (1) The sample was predominantly male (79.8%) with a mean age of 42 years. Extrapolation of the results to female, older adult, or adolescent OSA patients should be done with caution. Data from a single center may be subject to regional selection bias. (2) The 34-s/52-s thresholds were derived from a statistical model and need to be validated by prospective studies to determine if they can improve patient prognosis when used to guide clinical interventions such as CPAP titration. (3) The study focused on statistical associations rather than biological mechanisms. It did not analyze the physiological pathways through which LAD affects oxygenation (e.g., upper airway collapse patterns, ventilation/perfusion ratios). Further basic research is needed to supplement these findings. (4) While we used LAD as our primary exposure, it can theoretically be susceptible to rare artifacts. Future studies could consider using even more robust measures, such as 95% of the longest apnea duration, to corroborate our findings. (5) Future studies can explore how to incorporate baseline SaO_2_ to improve the prediction model for individual patients.

## 5 Conclusion

This study confirms that LAD is an independent risk factor driving LSaO_2_ decline in OSA patients and establishes 34-s and 52-s thresholds as early-warning indicators for moderate and severe hypoxia, respectively. These findings catalyze a paradigm shift in OSA evaluation—from event frequency (AHI) to event harmfulness (hypoxic depth). Although further validation is required regarding the generalizability of these thresholds and their association with hard endpoints, LAD as a key component of “hypoxic burden” holds promise as a novel biomarker for individualized risk stratification and targeted therapeutic interventions. Future research should integrate multidimensional hypoxic indicators (peak duration, cumulative burden, reoxygenation rate) to develop precision prediction models for OSA-related end-organ damage.

## Data Availability

The original contributions presented in the study are included in the article/supplementary material, further inquiries can be directed to the corresponding author.
